# Supplementary material for: Aβ42 oligomers trigger synaptic loss through CAMKK2-AMPK-dependent effectors coordinating mitochondrial fission and mitophagy
Source: Nat Commun. 2022 Aug 1;13:4444. doi: 10.1038/s41467-022-32130-5 (PMC9343354; doi:10.1038/s41467-022-32130-5)
Supplement: Supplementary file 1 — Supplementary Information [file 41467_2022_32130_MOESM1_ESM.pdf]

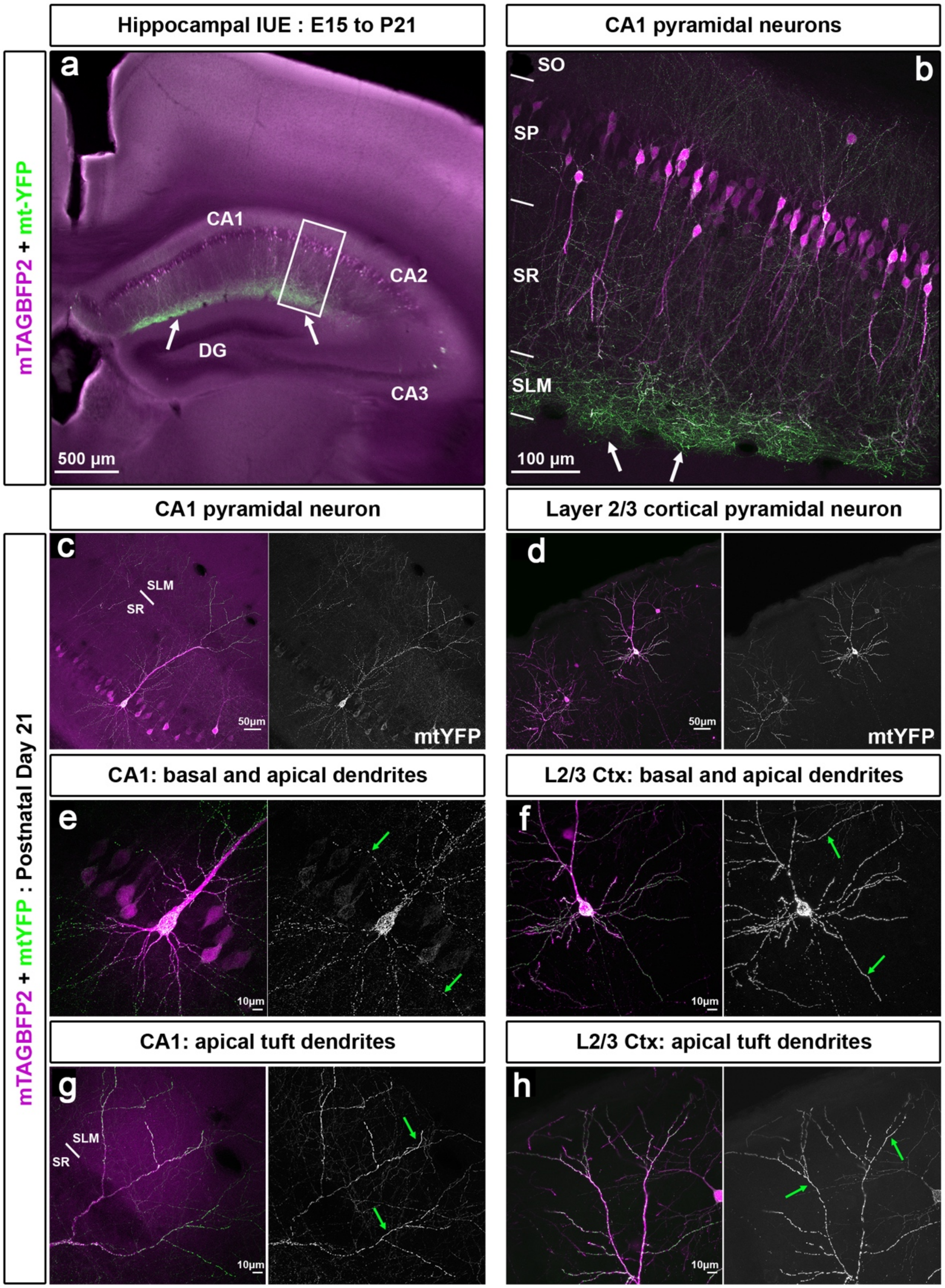

Figure S1. Hippocampal CA1 pyramidal neurons are characterized by highly compartmentalized dendritic mitochondria morphology compared to layer 2/3 cortical pyramidal neurons.

(**a**) Low magnification image of hippocampal CA1 pyramidal neurons *in utero* electroporated at E15.5 with pCAG-mTagBFP2 (purple) and pCAG-mitoYFP (green) imaged at P21. (**b**) High magnification image of inset in panel **a**, highlighting that the distal apical tuft dendrites in the SLM layer contain a high density of mitochondria compared to the basal and oblique dendrites (in SO and SR respectively). (**c**) Low magnification of a single optically isolated CA1 pyramidal neuron, and (**e**, **g**) high magnification of its proximal dendrites (basal and apical oblique in **e**) as well as apical tuft dendrites (panel **g**) highlighting that mitochondria in the basal and oblique dendrites are small and fragmented (green arrows in **e**) in proximal dendrites whereas mitochondria in apical tuft of the same CA1 PN are fused (green arrows in **g**). (**d**) Low magnification of a single optically isolated cortical layer 2/3 PN, and (**f**, **h**) high magnification images of its proximal dendrites (basal and apical oblique in panel **f**) and apical tuft (panel **h**), highlighting that mitochondria morphology is homogenously elongated and fused throughout the dendritic arbor (green arrows in **f**, **h**) of layer 2/3 PNs. Each image shown has been observed in at least 3 independent in utero electroporated brains and quantification of mitochondria morphology for CA1 hippocampal neurons is shown in Figure 1. Abbreviations: SLM: stratum lacunosum moleculare; SR: stratum radiatum; SO: stratum oriens; SP: stratum pyramidalis.

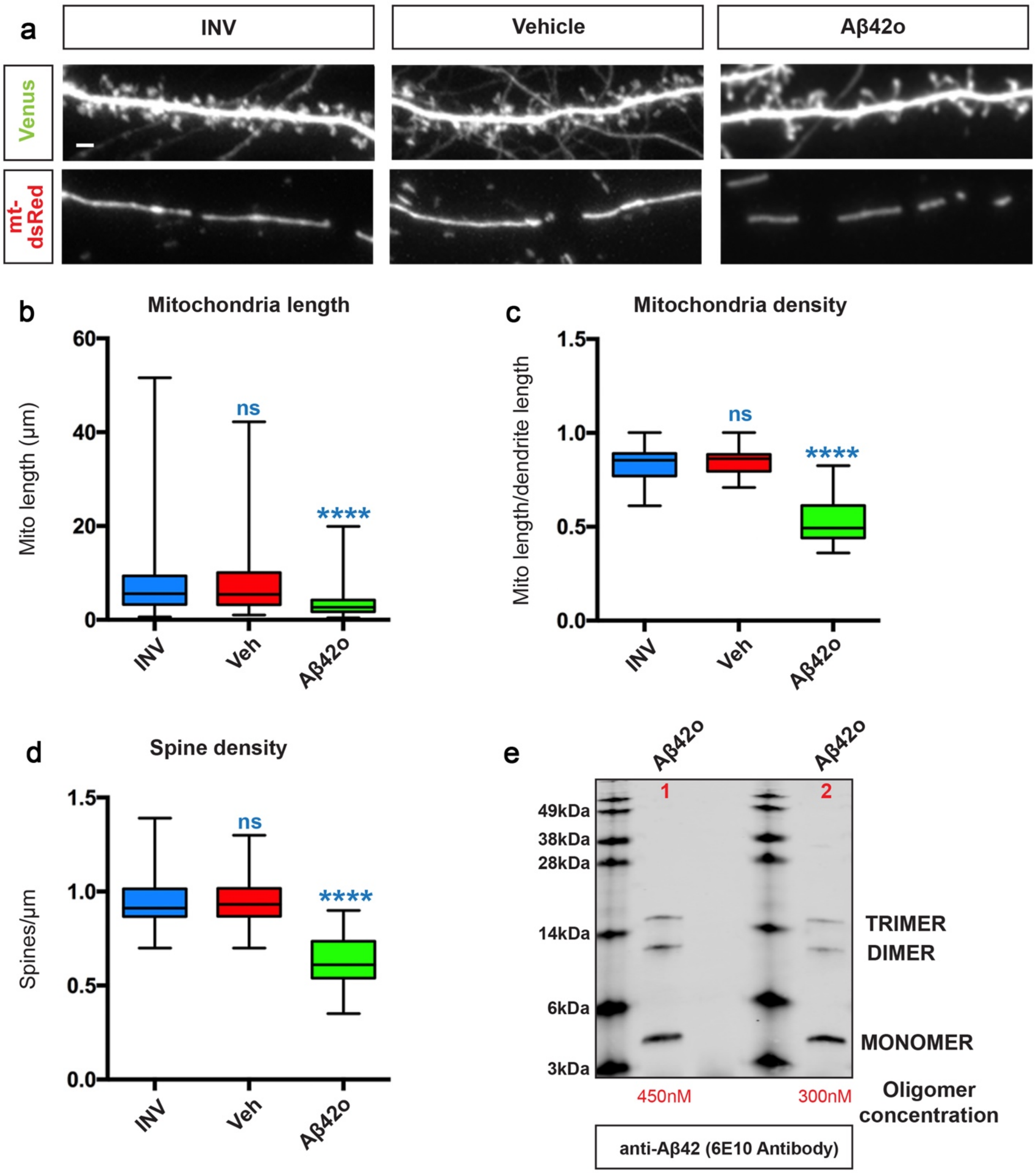

Figure S2. Treatment with control inverse oligomeric Aβ42 does not influence mitochondrial morphology or spine density.

(**a**) Representative high magnification images of dendritic segments showing dendritic spines in the upper panel and mitochondria in the lower panel. Treatment with inverse Aβ42o does not affect spine density or mitochondrial morphology, and similar to DMSO treated neurons. (**b**) Mitochondrial length for inverse Aβ42o is comparable to DMSO treated neurons, while Aβ42 oligomer treatment significantly reduces mitochondrial length. (**c**) Mitochondrial density for inverse Aβ42o is comparable to DMSO treated neurons

while Aβ42 oligomer treatment significantly reduces mitochondrial density. (**d**) Spine density quantification for inverse Aβ42o is comparable to DMSO treated neurons, while Aβ42 oligomer treatment significantly reduces spine density. All analyses were done blind to the experimental conditions, and were done by manual counting using FIJI. Data is represented by box plots displaying minimum to maximum values, with the box denoting $25^{th.}$, 50^{th}(median) and 75th percentiles from three independent experiments. n_{INV} = 41 dendrites, 243 mitochondria; n_{Veh} = 31 dendrites; 178 mitochondria; $n_{A\beta42o}$ = 33 dendrites, 245 mitochondria. Statistical analysis was performed using Kruskal-Wallis test followed by Dunn's post-hoc test in (**b-d**). The test was considered significant when $p<0.05$ with the following criteria: * $p<0.05$; ** $p<0.01$; ***$p<0.001$; ****$p<0.0001$; ns, not significant. Scale bar= 2μm. (**e**) 16.5% Tris-Tricine SDS-PAGE was performed to resolve Aβ42 oligomers from the peptide monomer that were generated as described in Material and Methods. Immuno-blotting was performed with 6E10 antibody which shows a mixture of monomers, dimers and trimers in lane one and two (two independent oligomerization experiments). For quantifying the relative oligomer/monomer concentration in lane one and two, near-infrared fluorescence signal intensity was measured for dimer plus trimer, and monomer using an Odyssey Imager. Optical density for Lane 1: Dimer+Trimer: 1510; Monomer: 1830. Signal intensity values for Lane 2: Dimer+Trimer: 736; Monomer: 1720.

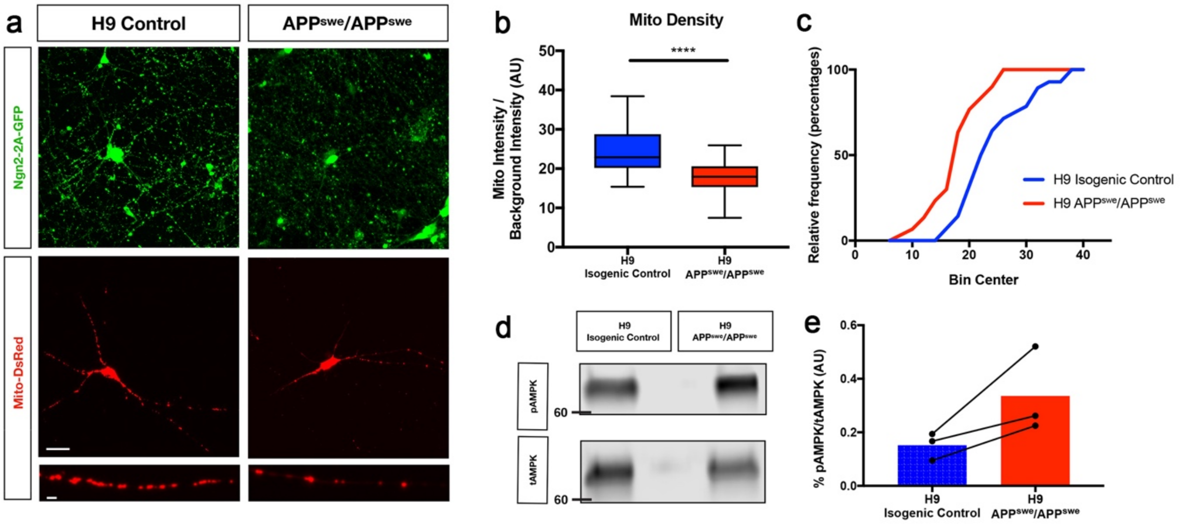

Figure S3. Human ES-derived cortical-like pyramidal neurons expressing endogenous APP^{swe} mutations exhibit decreases in mitochondrial density and increased AMPK activation.

(**a**) Representative low and high magnification images of neurons transdifferentiated from control human H9 embryonic stem (hES) cells and $APP^{swe/swe}$ knock-in hES cells at 24 DIV. hES-derived cortical-like neurons were induced using a Ngn2-dependent neural induction and were lipofected at 21DIV with a pCAG-mitoDsRed reporter gene to label mitochondria, fixed at 24 DIV, and imaged by confocal microscopy. Endogenous expression of the APP^{swe}/APP^{swe} mutation alone is sufficient to induce a significant decrease in mitochondrial density in their dendrites (bottom high magnification panels). (**b**) Quantification of neuritic mitochondrial density shows a significant decrease in the mitochondrial density in the H9 APP^{swe}/APP^{swe} cells compared to the H9 isogenic controls. All of the analyses were done by kymographic fluorescent density measurement in Nikon Elements software and blind to genotype. (**c**) Cumulative frequency distribution of mitochondrial density in Ngn2-induced cortical-like neurons derived from control isogenic H9-hES cells (blue) and APP^{swe}/APP^{swe} hES cells (red) confirms a decrease in mitochondrial density in cells expressing APP^{swe}/APP^{swe}. (**d**) Sample western blot of H9 isogenic control versus H9 APP^{swe}/APP^{swe} demonstrates an increase in phosphorylated AMPK in APP^{swe}/APP^{swe} expressing cells. 50µg of immunoprecipitated cell lysate was immunoblotted with phospho-AMPK (T172, CST) to confirm activation of AMPK under endogenous expression of APP^{swe} mutations. (**e**) Quantification of increased phospho-AMPK, representing mean with all three paired values represented by individual points. In panel b, data represented by box plots are displaying minimum to maximum values, with the box denoting 25th, 50th (median), and 75th percentiles from three independent experiments. In a-c: $n_{H9control}$ = 227 neurites, 70 neurons; n_{APPswe} = 203 neurites, 73 neurons. Statistical analyses in b were performed using one-way ANOVA followed by Kruskal-Wallis post-hoc. The test was considered significant when $p < 0.05$ with the following criteria: **** $p < 0.0001$; ns, not significant. Scale bars in panels a : low magnification panels: 20 microns; high magnification panels (bottom): 2 microns.

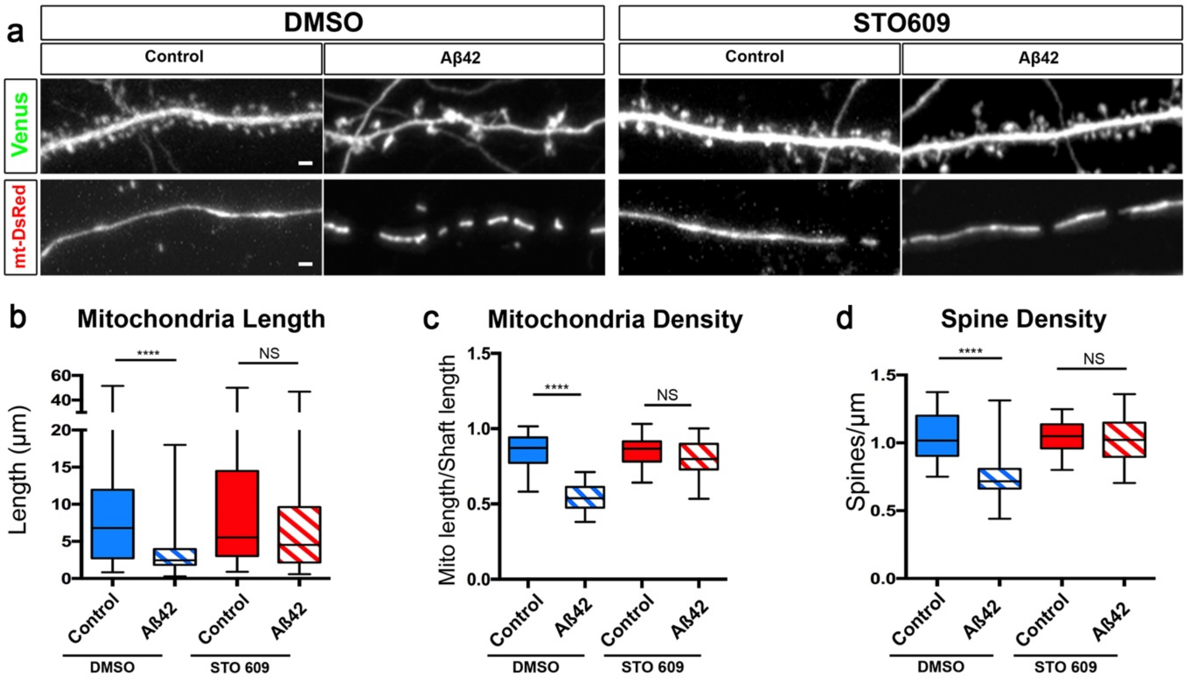

Figure S4. Oligomeric Aβ42 induced synaptotoxicity and dendritic mitochondrial fragmentation is CAMKK2 dependent.

(**a**) Secondary dendritic segments of primary cortical neuron at 21 DIV. Embryos at E15.5 were *ex utero* electroporated with pCAG-Venus and pCAG-mito-DsRed. At 20 DIV, neurons were pre-treated with either DMSO or STO609 (2.5μM) for 2 hours before being treated with either control or Aβ42 oligomers for 24 hours. (**b**) Quantification of dendritic mitochondrial length, (**c**) dendritic mitochondrial density, and (**d**) spine density show that blocking CAMKK2 activity via STO609 blocks both dendritic mitochondrial remodeling and synaptotoxicity.. All the analyses were done blind to the experimental conditions, and were done by manual counting using FIJI. Data is represented by box plots displaying minimum to maximum values, with the box denoting 25th, 50th (median), and 75th percentiles from three independent experiments. $n_{DMSO\ Control}$ = 37 dendrites, 163 mitochondria; $n_{DMSO\ A\beta42o}$ = 31 dendrites, 248 mitochondria; $n_{STO609\ Control}$ = 37 dendrites, 163 mitochondria; $n_{STO609\ A\beta42o}$ = 35 dendrites, 157 mitochondria. Statistical analyses were performed using Kruskal-Wallis test followed by Dunn's post-hoc test in (b-d). The test was considered significant when $p<0.05$ with the following criteria: * $p<0.05$; ** $p<0.01$; ***$p<0.001$; ****$p<0.0001$; ns, not significant. Scale bar = 2μm.

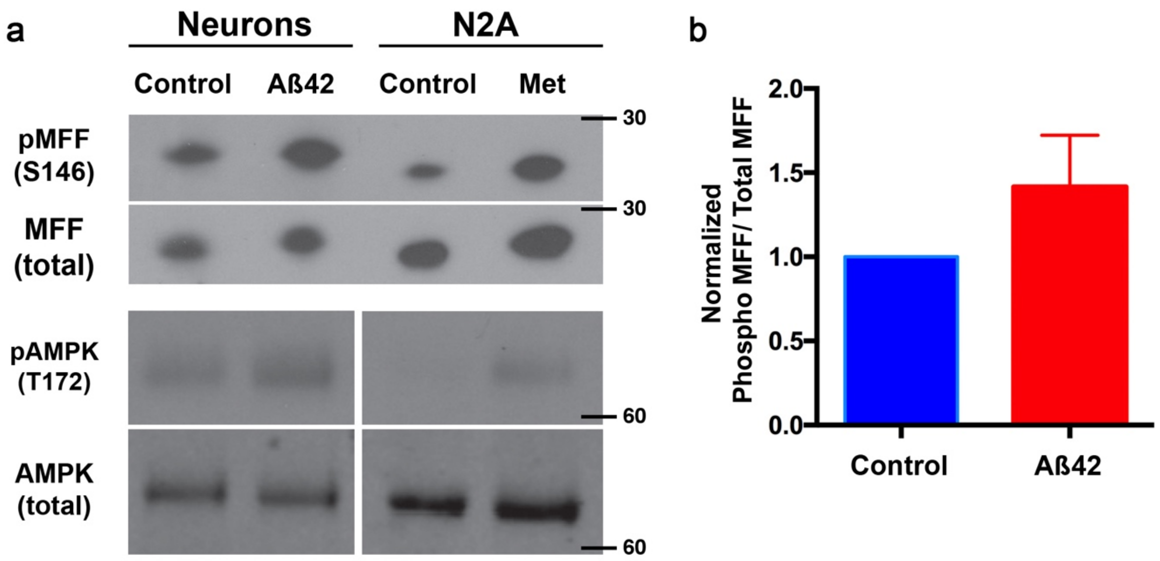

Figure S5. Aβ42o-dependent increase in MFF phosphorylation.

(**a**) Cortical PNs maintained in dissociated culture were treated at 20DIV with either a control vehicle or Aβ42o for 24 hours (left two lanes). N2A cells were treated with either DMSO or 2mM of Metformin for 5 hours as a positive control for AMPK-dependent MFF phosphorylation[2] (right two lanes). 80µg of whole cell lysate was immunoprecipitated with MFF antibody (Progen) and immunoblotted with either MFF antibody or phospho-MFF antibody (S146, CST). Whole cell lysates were also immunoblotted with either AMPK or phospho-AMPK (T172, CST) to confirm activation of AMPK under both Aβ42o for 24 hours [1] and metformin treatment for 5 hours [2]. (**b**) Quantification of immunoblot phospho-MFF band intensity normalized to total MFF levels demonstrate an increase in phospho-MFF in neurons treated with Aβ42o (n=3 independent cultures).

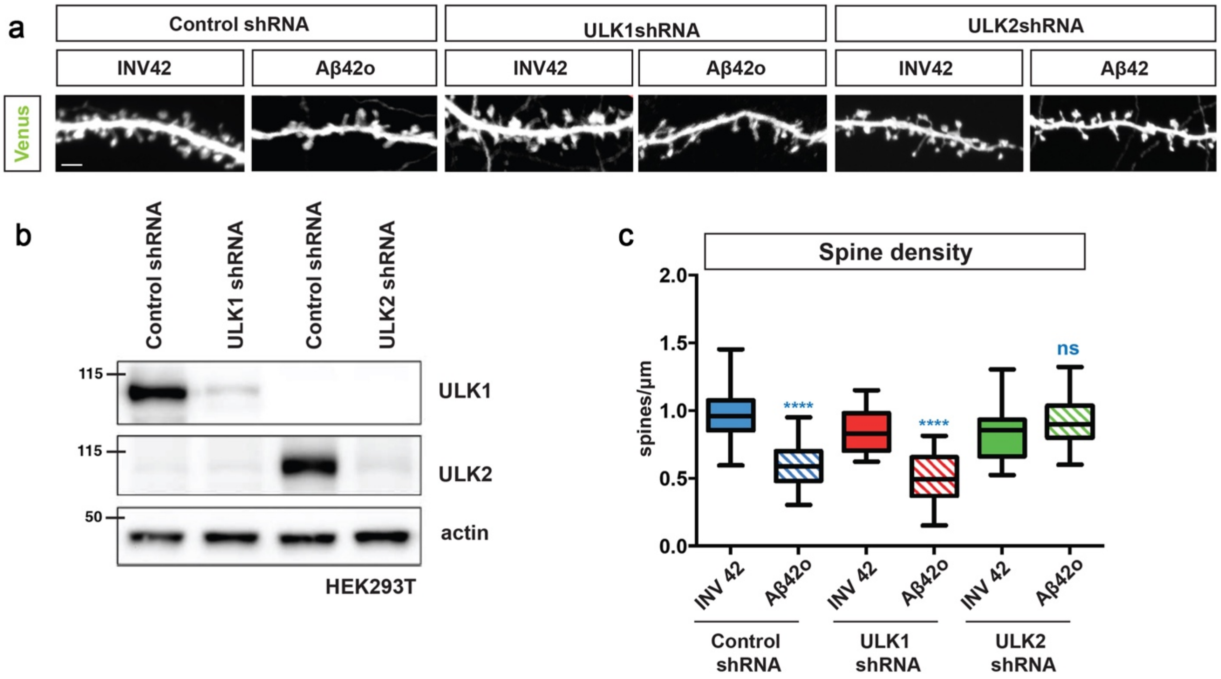

Figure S6. ULK2 but not ULK1 is required for Aβ42o-dependent synaptotoxic effects.

(**a**) Representative images of secondary dendritic segments of primary cortical PNs treated with either control or Aβ42o at 21DIV for 24 hours. Embryos were subjected to *ex utero* electroporation at E15.5 with pCAG-Venus and either a control shRNA or an shRNA specific for the kinase ULK1 or ULK2. Knockdown of ULK2, but not ULK1 kinase, blocks Aβ42o-induced synaptotoxicity. (**b**) Western blot showing validation of both the ULK1 and ULK2 shRNAs, and the specificity of the shRNAs for their respective isoform. HEK 293T cells were transiently co-transfected with either control shRNA or ULK1/ULK2 shRNA, along with myc-mULK1 or Flag-mULK2 respectively, and western blotting was performed with 25µg of lysate with the indicated antibody. The same results were obtained for three independent cell cultures. (**c**) Quantification of spine density for panel a. All of the analyses were done blind to the experimental conditions and were done by manual counting using FIJI. In panel c, data is represented by box plots displaying minimum to maximum values, with the box denoting 25th, 50th (median) and 75th percentile from three independent experiments. n_{PLKO} control = 31 dendrites; n_{PLKO} Aβ42o = 27 dendrites; $n_{ULK1shRNA\ Control}$ = 27 dendrites; $n_{ULK1shRNA\ A\beta42o}$ = 24 dendrites; $n_{ULK2shRNA\ Control}$ = 33 dendrites; $n_{ULK2shRNA\ A\beta42o}$ = 35 dendrites. Statistical analyses were performed using Kruskal-Wallis test followed by Dunn's post-hoc test in panel c. The test was considered significant when p<0.05 with the following criteria: * p<0.05; ** p<0.01; ***p<0.001; ****p<0.0001; ns, not significant. Scale bar = 2µm.

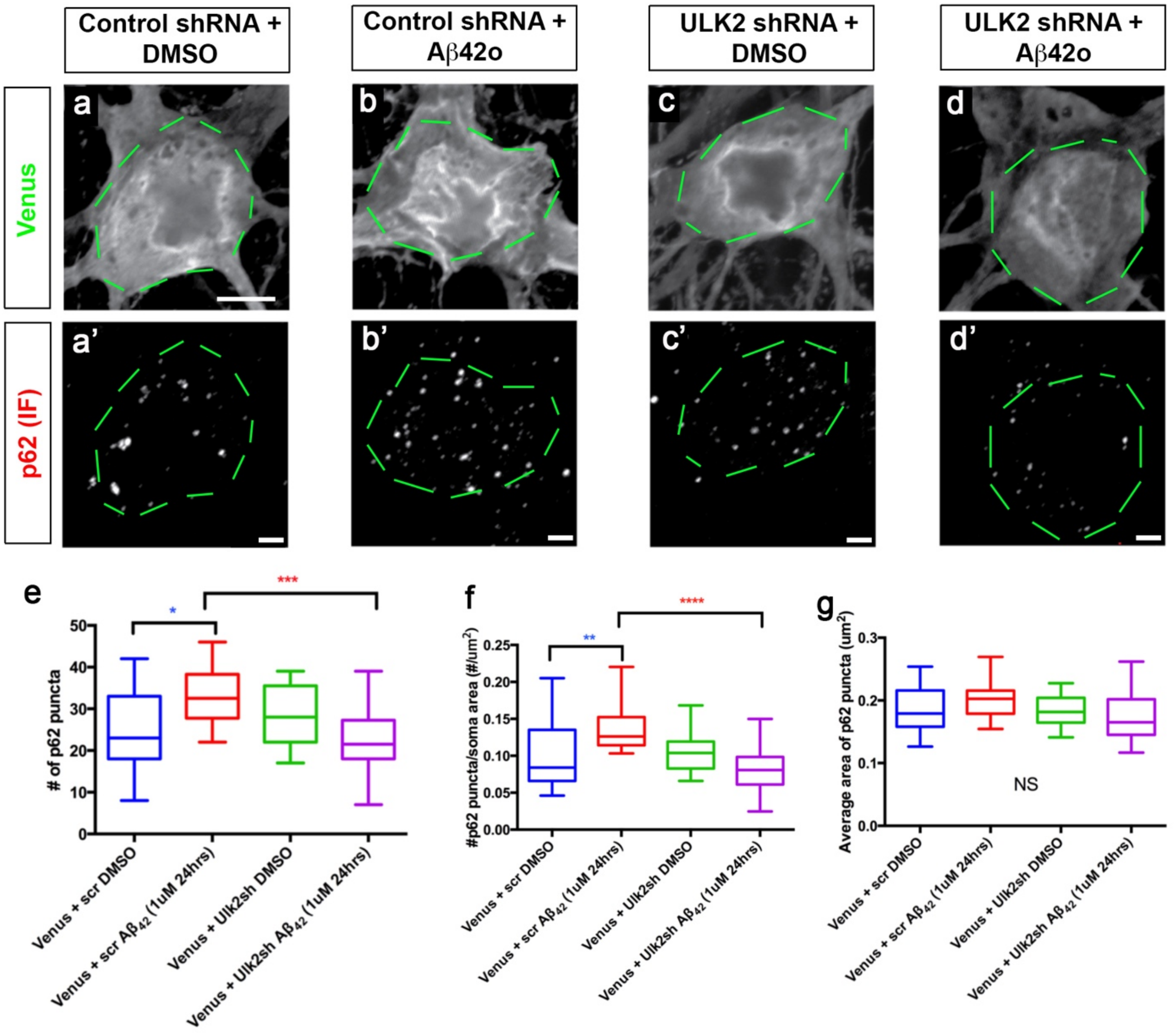

Figure S7. Knocking down ULK2 prevents the increased in p62 autophagosomes density induced by Aβ42 oligomers in cortical pyramidal neurons. (**a-d’**) Images of soma from cortical layer 2/3 PNs cultured for 21DIV following ex utero electroporation with Venus as a cell filler in combination with control (scrambled) shRNA (a-a’ and c-c’) or ULK2 shRNA (see Fig. 6B for validation) (b-b’ and d-d’). Cultures were treated for 24h prior to fixation with either vehicle (DMSO) or Aβ42 oligomers prepared as in Fig. S1. Following fixation, cultures were stained by immunofluorescence for p62 (also called SQSTM1), a ubiquitin-binding protein degraded marking autophagosomes. (**e-g**) Quantification of number of p62 puncta per soma (e), number of p62+ puncta normalized to soma area (f) and area of p62 puncta found over soma (g). In panels e-g, data is represented by box plots displaying minimum to maximum values, with the box denoting 25th, 50th (median) and 75th percentile from three independent experiments. Each experiment quantified was performed in triplicate of independent cultures. Number of cells analyzed: $n_{Venus+control\ shRNA+DMSO}$ =21; $n_{Venus+control\ shRNA+A\beta42o}$=18; $n_{Venus+ULK2shRNA+DMSO}$=21; $n_{Venus+ULK2\ shRNA+A\beta42o}$=22. Statistical analysis was performed using Kruskal-Wallis test followed by Dunn’s post test. * $p<0.05$ **$p<0.01$; *** $p< 0.005$; ****$p<0.001$. Scale bars in a-d’: 2 microns.

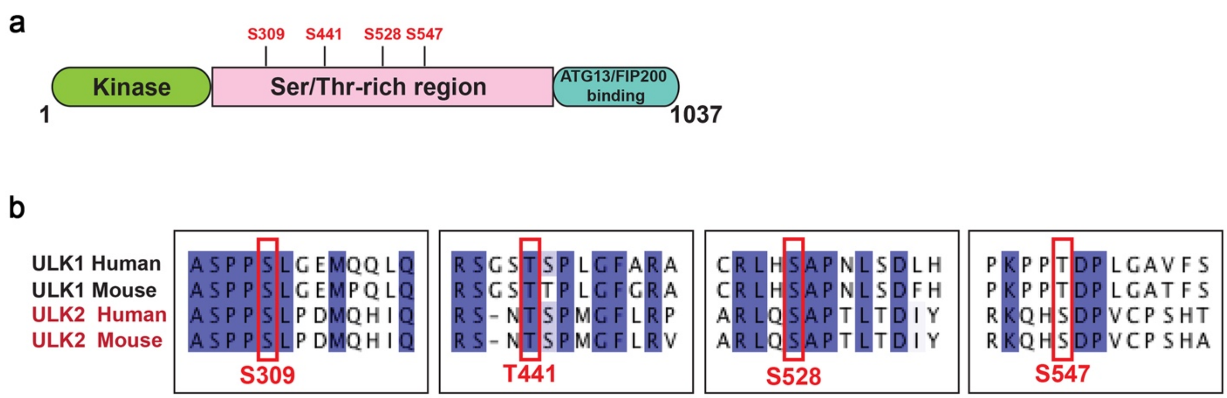

Figure S8. Schematic of conserved AMPK-mediated phosphorylation sites in ULK2.

(**a**) Schematic of mULK2 domain structure highlighting the four predicted AMPK-mediated phosphorylation sites. (**b**) ClustalW multiple sequence alignment of human and mouse ULK1 and ULK2 showing a high degree of conservation of four AMPK-mediated phosphorylation sites in ULK2 which were previously reported in ULK1[3].

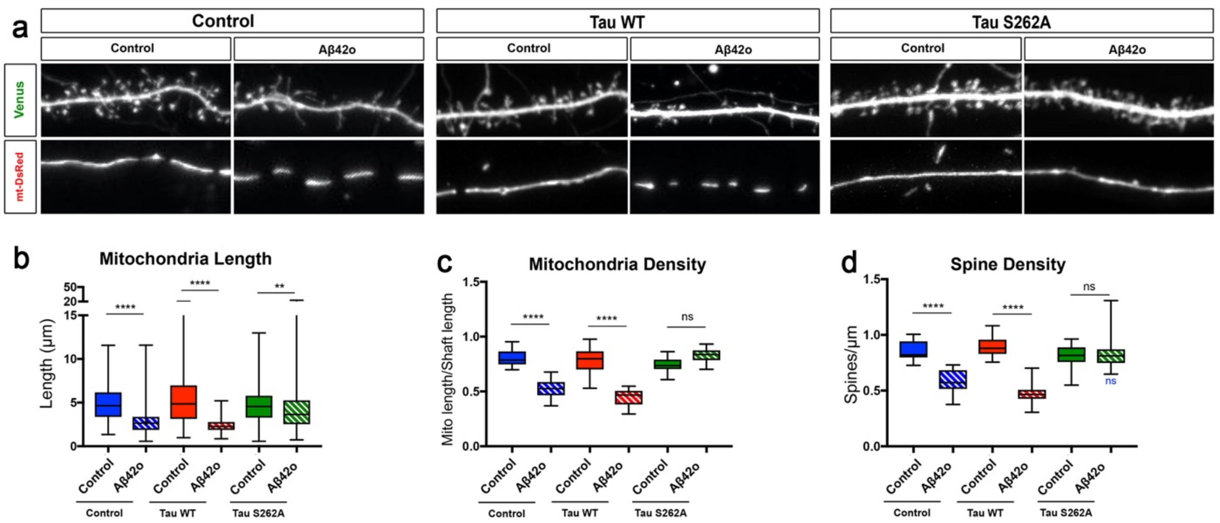

Figure S9. Tau phosphorylation at S262 is required for Aβ42o-induced dendritic mitochondrial remodeling.

(**a**) Representative images of secondary dendritic segments of primary cortical PNs at 21 DIV. E15.5 mouse embryos were *ex utero* electroporated with mVenus, mitoDsRed only or in combination with either wildtype hTau (isoform 4R2N) or a non-phosphorylatable mutant hTau-S262A. Neurons were treated at 20 DIV with either a vehicle or Aβ42o for 24 hours. Overexpression of hTau in the presence of Aβ42o decreases spine density, mitochondrial length, and mitochondrial density, while over expression of hTauS262A blocked oligomer-induced spine loss and mitochondrial density decrease, but not mitochondrial fragmentation. (**b**) Quantification of dendritic mitochondrial length, (**c**) dendritic mitochondrial density, and (**d**) dendritic spine density. All of the analyses were done blind to the experimental conditions and were done by manual counting in FIJI. In panels b-d, data is represented by box plots displaying minimum to maximum values, with the box denoting 25th, 50th (median) and 75th percentile from three to six independent experiments. $n_{control}$= 81 dendrites, 593 mitochondria; $n_{control\ A\beta42o}$ = 105 dendrites, 645 mitochondria; $n_{hTauWT\ Control}$ = 38 dendrites, 258 mitochondria; $n_{hTauWT\ A\beta42o}$ = 79 dendrites, 725 mitochondria; $n_{hTauS262A\ Control}$ = 81 dendrites, 555 mitochondria; $n_{hTauS262A\ A\beta42o}$ = 89 dendrites, 756 mitochondria. Statistical analyses were performed using One-way ANOVA followed by Kruskal-Wallis Multiple Comparisons in (b-d). The test was considered significant when $p < 0.05$ with the following criteria: * $p < 0.05$; ** $p < 0.01$; *** $p < 0.001$; **** $p < 0.0001$; ns, not significant.

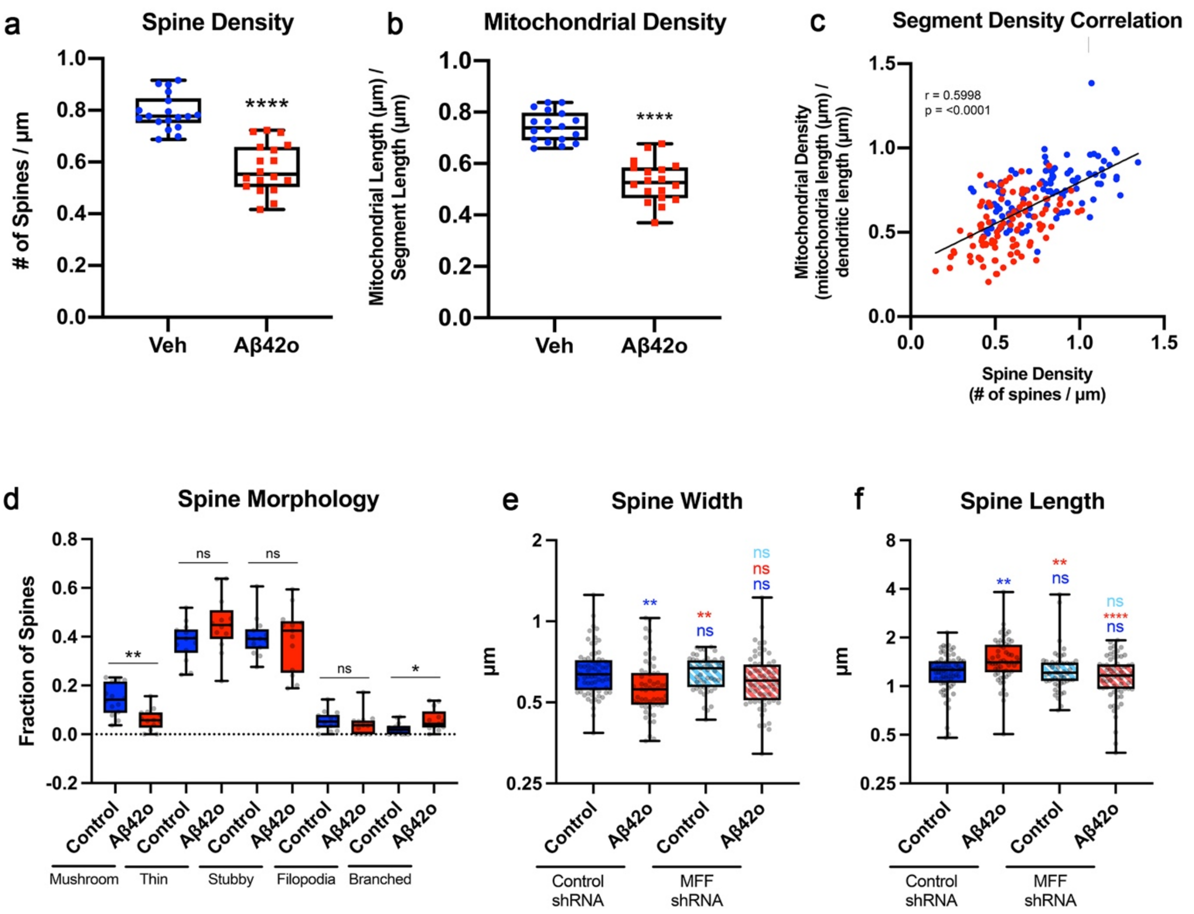

Figure S10. Correlation between degree of dendritic mitochondrial remodeling and spine densities and morphological features of dendritic spines induced by Aβ42o are rescued by MFF knockdown.

(**a-b**) Quantification spine density (a) and mitochondrial density (b) in secondary dendritic segments of primary cortical PNs at 21DIV following 24 hours of treatment with either a vehicle control (blue) or Aβ42o (red values). (**c**) Correlation between dendritic mitochondrial density (volume of dendrite occupied by mitochondria) and spine density in individual secondary dendritic segments treated with either a vehicle control (blue points) or Aβ42o (red points). Non-parametric Spearman correlation coefficient (r) is highly significant. (**d**) Categorization of spine morphology for primary cortical PNs treated with either a vehicle control or Aβ42o. (**e-f**) Quantification of dendritic spine width (e) and dendritic spine width (f) for primary cortical PNs *ex utero* electroporated with pCAG-mVenus, pCAG-mito-DsRed, and either a control shRNA or MFF shRNA, treated with either a vehicle control or Aβ42o. In all panels (except panel c), data is represented by box plots displaying minimum to maximum values, with the box denoting 25th, 50th (median), and 75th percentile, with each data point representing an individual dendritic segment. Statistical analyses were performed using a Mann-Whitney test. The data was quantified using three to six biological replicates, n_{Veh} = 99 dendritic segments, 750 mitochondria; $n_{A\beta42o}$ = 101 dendritic segments, 840 mitochondria; $n_{ControlshRNA\ Veh}$ = 13 dendritic segments, 600 spines ; $n_{ControlshRNA\ A\beta42o}$ = 12 dendritic segments, 490 spines; $n_{MFFshRNA\ Veh}$ = 8 dendritic segments, 360 spines ; $n_{MFFshRNA\ A\beta42o}$ = 9 dendritic segments, 394 spines. All of the analyses were done blind to the experimental conditions, using dendritic segments of similar length (43µm on average), and were done by manual counting using Fiji. The tests were considered significant when p<0.05 with the following criteria: *p<0.05; ** p<0.01, *** p<0.001, **** p<0.0001.

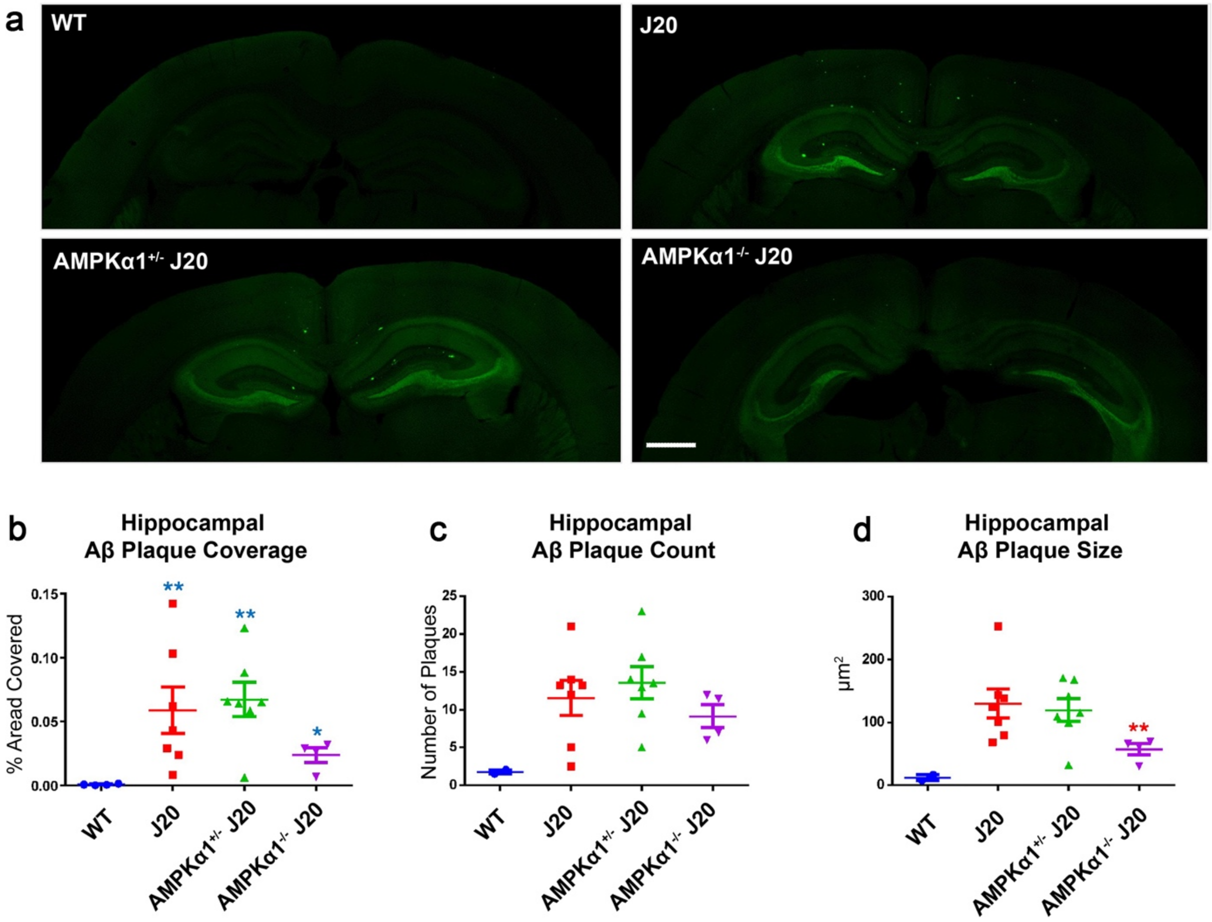

Figure S11. Aβ plaque load is decreased in the hippocampus of 5 months-old AMPKα1$^{-/-}$ J20 mice. (**a**) Coronal sections from 5 month-old WT, J20 (hets), AMPKα1$^{+/-}$(HT)xJ20(hets) and AMPKα1$^{-/-}$(KO)xJ20(hets) mice immunolabeled with 6E10 antibody (detecting Aβ42 oligomers) reveals amyloid plaques. (**b**) The area covered by amyloid plaques, (**c**) the number of amyloid plaques, and (**d**) their size were measured in the hippocampus. These quantifications suggest that Aβ plaque coverage and Aβ plaque size is reduced in the J20 mouse when AMPKα1 expression is reduced in a dose-dependent manner, although the number of plaques does not seem to be decreased. Statistical analyses were performed using Mann-Whitney test between each genotype versus WT (blue stars) or J20 (red stars). In panels b-d, bar graph represent mean +/- S.E.M. The tests were considered significant when $p<0.05$ with the following criteria: * $p<0.05$; ** $p<0.01$. n = 4-7 animals per genotype. Scale bar in panels a: 200 microns.

Supplementary Movie S1. Time-lapse of DIV 21 Inverse peptide treated cortical pyramidal neurons expressing mito-mTagBFP2, LAMP1-mEmerald, and RFP-LC3 to visualize mitochondria, lysosomes, and autophagosomes respectively. The dendritic segment corresponds to the representative image in Figure 3. Neurons were imaged every 15 minutes for 14 hours.

Supplementary Movie S2. Time-lapse of DIV 21 Aβ42o treated cortical pyramidal neurons expressing mito-mTagBFP2, LAMP1-mEmerald, and RFP-LC3 to visualize mitochondria, lysosomes, and autophagosomes respectively. Dendritic segment corresponds to the representative image in Figure 3. Neurons were imaged every 15 minutes for 14 hours.

Supplementary References

1 Mairet-Coello, G. *et al.* The CAMKK2-AMPK kinase pathway mediates the synaptotoxic effects of Abeta oligomers through Tau phosphorylation. *Neuron* **78**, 94-108, doi:10.1016/j.neuron.2013.02.003 (2013).

2 Toyama, E. *et al.* AMP-activated protein kinase mediates mitochondrial fission in response to energy stress. *Science* **351**, 275-281 (2016).

3 Egan, D. F. *et al.* Phosphorylation of ULK1 (hATG1) by AMP-activated protein kinase connects energy sensing to mitophagy. *Science* **331**, 456-461, doi:10.1126/science.1196371 (2011).
