# Supplementary material for: Aβ42 oligomers trigger synaptic loss through CAMKK2-AMPK-dependent effectors coordinating mitochondrial fission and mitophagy
Source: Nat Commun. 2022 Aug 1;13:4444. doi: 10.1038/s41467-022-32130-5 (PMC9343354; doi:10.1038/s41467-022-32130-5)
Supplement: Supplementary file 3 — Description of Additional Supplementary Files [file 41467_2022_32130_MOESM3_ESM.pdf]

Title: Supplementary Movie 1.
Description: Time-lapse of DIV 21 Inverse peptide treated cortical pyramidal neurons expressing mito-mTagBFP2, LAMP1-mEmerald, and RFP-LC3 to visualize mitochondria, lysosomes, and autophagosomes respectively. The dendritic segment corresponds to the representative image in Figure 3. Neurons were imaged every 15 minutes for 14 hours.

Title: Supplementary Movie 2.
Description: Time-lapse of DIV 21 Aβ42o treated cortical pyramidal neurons expressing mito-mTagBFP2, LAMP1-mEmerald, and RFP-LC3 to visualize mitochondria, lysosomes, and autophagosomes respectively. Dendritic segment corresponds to the representative image in Figure 3. Neurons were imaged every 15 minutes for 14 hours.
